# Comparison of minimally invasive percutaneous fixation and open reduction internal fixation for patella fractures: a meta-analysis

**DOI:** 10.1186/s13018-021-02612-1

**Published:** 2021-08-17

**Authors:** Chun-Hong Lo, Chih-Hwa Chen

**Affiliations:** 1 Department of Primary Medicine, Taipei Medical University Shuang Ho Hospital, New Taipei City, Taiwan; 2 Department of Orthopedics, Taipei Medical University Shuang Ho Hospital, New Taipei City, Taiwan; 3 School of Biomedical Engineering, College of Biomedical Engineering, Taipei Medical University, Taipei, Taiwan; 4 Research Center of Biomedical Device, Taipei Medical University, Taipei, Taiwan; 5 School of Medicine, College of Medicine, Taipei Medical University, Taipei, Taiwan

**Keywords:** Patella fractures, Minimally invasive, Percutaneous fixation, Open reduction internal fixation, Meta-analysis

## Abstract

**Background:**

Open reduction internal fixation (ORIF) has long been the conventional procedure for managing displaced patella fracture. This surgical approach has certain drawbacks, which might affect clinical outcomes and patient prognosis. Minimally invasive percutaneous fixation (MIPF) was proposed to overcome these disadvantages. Few in-depth investigations have been performed to determine the superiority of MIPF over ORIF. The aim of this study was to compare the efficacies of MIPF and ORIF for patella fractures.

**Methods:**

The PubMed, Cochrane Library, Embase, and Scopus databases were searched for relevant studies from November 26 to December 17, 2020. Non-English publications and pediatric orthopedic articles were excluded. Statistical analysis was performed using Review Manager, version 5.4, with mean differences (MDs), standardized mean differences (SMDs), odds ratios (ORs), and respective 95% confidence intervals (CIs) calculated using a random effects model. The primary outcomes were the pain score, knee range of motion, and joint functionality. The secondary outcomes were the surgical time, complications, and implant removal rate.

**Results:**

Six articles with a total of 304 patients were included in the meta-analysis. Pooled analysis revealed that patients with MIPF had a significantly reduced pain score (MD = − 1.30, 95% CI = − 1.77 to −0.82; *p* < 0.00001) and increased knee extension angles (MD = 0.72, 95% CI = 0.18 to 1.25; *p* = 0.009) at 3-month follow-up. Furthermore, knee flexion angles (MD = 8.96, 95% CI = 5.81 to 12.1; *p* < 0.00001) and joint functionality (SMD = 0.54, 95% CI = 0.21 to 0.86; *p* = 0.001) had statistically improved at 2 years. However, no difference was observed between MIPF and ORIF with regard to the surgical time. The risk of complications (OR = 0.10, 95% CI = 0.05 to 0.18; *p* < 0.00001) and implant removal rate (OR = 0.20, 95% CI = 0.07 to 0.57; *p* = 0.003) were significantly lower with MIPF than with ORIF.

**Conclusions:**

MIPF is more favorable than ORIF in terms of the pain score, knee range of motion, joint functionality, complications, and implant removal rate. Thus, it can be adopted as an alternative to ORIF.

**Supplementary Information:**

The online version contains supplementary material available at 10.1186/s13018-021-02612-1.

## Introduction

Patella, the largest sesamoid bone in the human body, serves as the fulcrum of our extensor apparatus [1]. Its fractures account for approximately 0.5–1.5% of all musculoskeletal injuries [2]. Surgery is indicated in case of displacement/articular step-off of > 2 mm, diastasis/fragment separation of > 3 mm, osteochondral fracture accompanied by intra-articular loose bodies, or an impaired extensor mechanism [3]. Although patella fractures are relatively uncommon, they are always a great challenge for orthopedic surgeons in terms of precise anatomical reduction, rigid fixation, and postoperative wound care [4].

Open reduction internal fixation (ORIF) has long been the mainstream treatment for displaced patella fractures. This surgical approach, however, has numerous disadvantages, which may disrupt its clinical outcome. These include the requirement for a long incision for direct visualization, causing substantial soft tissue compromise at the fracture site, potential devascularization of individual bone fragments, lengthened rehabilitation period, and heavy blood loss in relation to the dissection [5–10]. Complications, such as infection, delayed wound healing, irritation, or wire breakage, have been frequently reported in studies [11–13], with the incidence rate of symptomatic hardware being as high as 60% [3, 14].

In recent decades, minimally invasive percutaneous fixation (MIPF) has been proposed to minimize the aforementioned drawbacks. It is an indirect or limited open reduction technique involving small incisions, and it provides absolute stability through the use of cannulated screws, Kirschner wires, pins, or other implants inserted percutaneously [15]. Several modifications of this surgical method have been presented. Some studies have suggested novel fixation methods [16, 17], whereas the majority have advocated conducting the procedure with the assistance of arthroscopy and fluoroscopy [18–20]. So far, MIPF is not widely adopted due to the paucity of research on its benefits, complications, and biomechanical properties. The lack of a comparison between MIPF and ORIF may hinder physicians’ interest to adopt MIPF, and robust evidence regarding the superiority of MIPF is needed.

The objective of this meta-analysis was to compare the efficacies of MIPF and ORIF for patella fractures. We aimed to determine whether MIPF has superior outcomes through detailed statistical analysis.

## Methods

### Data sources and search strategy

PubMed, Cochrane Library, Embase, and Scopus databases were searched for relevant articles from November 26 to December 17, 2020. The following combinations of keywords were used: “patella (or kneecap) fracture*” and “minimally invasive or minimally invasive osteosynthesis (or surgery* or procedure*) or MIS or arthroscopic (arthroscopic-assisted)” or “percutaneous fixation (or osteosynthesis or pinning) or osteosynthesis,” and “open surgery (or reduction) or ORIF or tension band or K (or Kirschner) wire.”

Relevant Medical Subject Heading terms were used for searching PubMed and Cochrane Library databases. The titles and abstracts of all the relevant papers were skimmed to check their validity. In case of uncertainty regarding their relevance, the papers were read in detail. References in the selected articles were manually reviewed to identify potentially relevant articles. Duplications were excluded, and inclusion and exclusion criteria were applied. The latest article collected was published in 2020.

Only studies evaluating the outcomes of MIPF versus ORIF for patella fractures were included. They were selected based on clear inclusion and exclusion criteria, surgical techniques, follow-up period, and postoperative clinical evaluation of participants. The fixation tools used were not considered in this meta-analysis. Comparison studies that used different instruments were considered, but not studies in which two distinct operation methods were investigated. No restrictions were applied on the journal type, article type, or date of publication.

Articles that were merely abstracts, protocols, conference papers, animal models, and cadaver studies were excluded. Furthermore, articles with incomplete data, missing control group, and inconsistent methodology were excluded. Moreover, articles published in non-English language or those involving under-aged patients were excluded.

Two reviewers were responsible for selecting articles in accordance to the aforementioned criteria. Study and trial characteristics, such as experimental design, demographics, intervention, outcome measurement, and follow-up period, were abstracted independently. The reviewers’ decisions were subsequently compared, and discrepancies, if any, were resolved through in-depth discussions. Authors of the included articles were contacted through email for additional information when necessary.

### Methodological quality appraisal

The methodological quality of the included articles was assessed using the revised Cochrane risk-of-bias tool for randomized trials (RoB2) [21] and Risk Of Bias In Nonrandomized Studies–of Interventions (ROBINS-I) [22]. RoB2, which was specifically designed to assess randomized controlled trials (RCTs), contains a series of domains that evaluate selection, performance, attrition, detection, and reporting biases. ROBINS-I, which was designed for nonrandomized studies, consists of domains for evaluation of biases due to confounding, selection of participants into the study, classification of interventions, deviations from intended interventions, missing data, measurement of outcomes, and selection of reported results. Again, the assessment was performed separately by two reviewers. Any disagreement was resolved academically.

### Data extraction

After study selection, data regarding the clinical parameters of the MIPF and the open surgery groups were extracted. Number of patients, age, fracture type, time lag between injury and operation, surgical time, and functional assessment scores were recorded. All these data were obtained by the two aforementioned reviewers; the assessment procedure was the same as described above.

### Outcomes

The primary outcomes were the pain score, expressed in visual analog scale (VAS); knee flexion and extension angles; and joint functionality according to the Lysholm knee scoring system, Bostman clinical grading scale, or postoperative Knee Society Clinical Rating Scale. The secondary outcomes were the surgical time, complications, and implant removal rate. The 24-month follow-up data used in this meta-analysis included the data obtained at the final follow-up provided that the length of follow-up exceeded 24 months, and that there was no information reporting at the requiring time point.

### Data synthesis and statistical analysis

The meta-analysis was conducted using Review Manager, version 5.4 (Cochrane Collaboration, Oxford, England) according to Preferred Reporting Items for Systematic Reviews and Meta-Analyses guidelines [23]. Mean difference (MD) was used to evaluate the pain score, range of motion, and surgical time, whereas odds ratio (OR) was used to compare the complications and implant removal rates. To compare joint functionality, the results expressed using different scales were pooled for calculation using standardized mean difference (SMD). If joint functionality was unreported in the original article, standard deviations were estimated based on the provided confidence interval (CI) and standard errors. Unless otherwise specified, the precision of all pooled statistics was set to 95% CI, and statistics were analyzed using the inverse variance method with random effects model. For assessing statistical heterogeneity, the *I*^2^ test was performed. Outcome variability was considered high when the *I*^2^ value was > 75%, and sensitivity analysis was performed successively to confirm the test results after arithmetical adjustment. Subgroup analysis might be performed for improved interpretation, as needed. A *p* value of < 0.05 in the Cochran’s Q test implied significant heterogeneity.

## Results

### Study selection

**Fig. 1:**
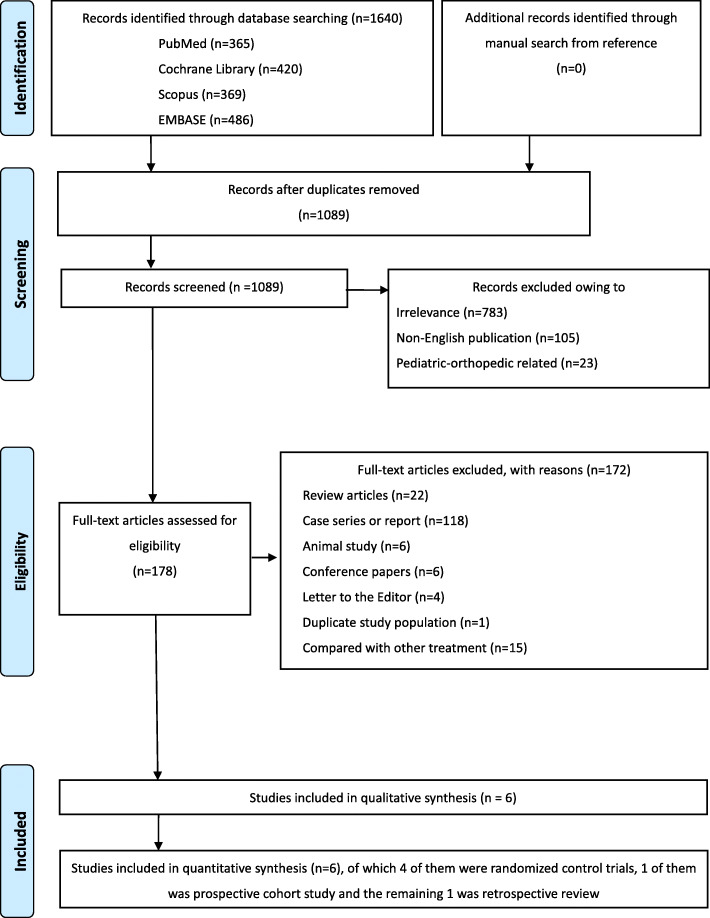
Flowchart of article selection

The database search retrieved 1640 potentially relevant articles. Of these, 551 duplicates were removed, and the remaining articles were assessed further. Of the remaining 1089 articles, 911 were ineligible as they were irrelevant to our research topic, non-English publications, or pediatric orthopedic studies. After full-text evaluation of the 178 articles, 172 were excluded for various reasons. Finally, 6 articles [10, 24–28] were found appropriate for this meta-analysis. The detailed selection flowchart is presented in Fig. 1.

### Study characteristics

The aforementioned articles were published between 2006 and 2020. The selected articles consisted of four RCTs, one prospective cohort study, and one retrospective review. The study sample sizes ranged from 38 to 60, with a total of 304. One study [10] involved patients aged > 60 years; one study [26] specifically limited their targeted population to the 18–65-year age group. These studies were slightly different from the other studies that had participants aged > 16 [24] or 18 years in general [25, 27, 28]. All articles, except two [25, 27], applied the explicit inclusion criterion of transverse patella fracture with displacement > 3 mm. All the articles had the uniform exclusion criteria of (1) open injury and (2) comminuted or multiple fractures. Three articles [24, 26, 28] additionally excluded multiple traumas; previous chronic degenerative disease, such as knee osteoarthritis and its associated fracture; previous surgical intervention of the knee; peripheral neural damage; uncompensated diabetes; and severe osteoporosis. All the studies compared MIPF with ORIF. Patella fractures with Arbeitsgemeinschaft fur osteosynthesefragen/Orthopaedic Trauma Association classification 34C1 or 45C1-3 were treated. Their outcome parameters were mostly the same, but their follow-up intervals had slight differences in accordance to the study design. Bostman clinical grading scale and the Lysholm knee scoring system were adopted by five studies, whereas one study [24] used the Knee Society Clinical Rating Scale as the functional evaluating tool. Rehabilitation protocols varied widely. The majority of them allowed passive range of motion and partial weight-bearing within 3 days postoperation. An articular knee brace was used in one trial [10], whereas the others had no external immobilizers at all times. One study [24] executed a relatively consistent rehabilitation plan instead of progressive mobilization training. Any complications were recorded in all selected articles. Participants were subjected to conservative treatment with or without reoperation. Study characteristics are summarized in Table 1.

**Table 1:** Characteristics of the selected articles

Study	Article type	No. of patients (Male/Female)	Age (years)	Type of fracture according to AO/OTA	Intervention	Outcomes	Functional evaluating scale	Follow-up (months)	Rehabilitation
Vicenti et al. (2020) [10]	Prospective study	S: 31 (12/19) C: 30 (13/17)	S: 69.62 ± 9.88 C: 70.77 ± 8.22	34-C1	S: MIOT with stainless steel wire C: OS with tension band wiring	abcde	Lysholm	1, 3, 6, 12, and 24	Postoperation day 1: Passive knee motion by using specific electric devices, progressive active mobilization, and static isometric quadriceps exercises + partial weight-bearing with crutches and brace (removed when satisfactory quadricep control was achieved)
Shao et al. (2019) [28]	RCT	S: 21 (14/7) C: 17 (11/6)	S: 42.2 ± 12.4 C: 40.3 ± 10.5	NM	S: MIS with cable pin system C: OS with cable pin system	abcde	Bostman	1, 2, and 12	Postoperation day 1: Passive exercise by using a continuous passive motion machine for three 1-h sessions, starting from 0^o^ to 60^o^, increasing 15^o^ per day until 90^o^ was achieved ± active flexion exercises in prone position Postoperation day 3: Partial weight-bearing 3 weeks postoperation: Active extension Radiographically healed: Full weight-bearing
Lin et al. (2015) [27]	RCT	S: 26 (15/11) C: 26 (13/13)	S: 50.8 ± 16.3 C: 52.5 ± 17.4	45-C1.1 45-C1.2 45-C1.3	S: CRCF C: ORTF	abcefg	Lysholm	3, 6, and 12	Quadricep–femur contraction excises soon after the operation + passive ROM under tolerable wound pain 3 weeks postoperation: Active ROM 8 weeks postoperation: Full weight-bearing
Mao et al. (2013) [26]	RCT	S: 20 (14/6) C: 20 (11/9)	S: 40.2 ± 10.0 C: 43.5 ± 11.4	NM	S: MICP C: OSKW	abcdef	Bostman	1, 3, 6, 12, and 24	Postoperation day 1: Passive exercise by using a continuous passive motion machine ± active flexion exercises in prone position Postoperation day 3: Partial weight-bearing 3 weeks postoperation: Active extension Radiographically healed: Full weight-bearing
Chiang et al., (2011) [20, 25]	Retrospective review	S: 20 (9/11) C: 40 (15/25)	S: 56.6 ± 14.7 C: 60.2 ± 15.4	45-C1.1 45-C1.3	S: POMC C: OMATB	bcdefg	Lysholm	1, 3, 6, 12, and 24	Postoperation day 1: Partial weight-bearing ± passive ROM if pain can be tolerated 3 weeks postoperation: Active ROM 8 weeks postoperation: Full weight-bearing
Luna-Pizarro et al. (2006) [24]	RCT	S: 27 (17/10) C: 26 (13/13)	S: 51 ± 14.8 C: 44 ± 18.2	45-C1.1 45C1.3	S: PPOS C: OS with modified tension band	abcdef	KSCRS	1, 2, 12, and 24	12 h postoperation: Isometric and isotonic contractions of quadriceps for 30 min four times a day and continued after discharge

*AO/OTA*, arbeitsgemeinschaft fur osteosynthesefragen/ orthopaedic trauma association; a, pain score; *Bostman*, Bostman clinical grading scale; b, knee range of motion (flexion/extension); *C*, control group; *CRCF*, closed reduction and percutaneous cannulated screw fixation; c, joint functionality; d, operation time; e, incidence of complications; f reoperation rate; ^g^ union time; *KSCRS* Knee Society Clinical Rating Scale, *Lysholm* Lysholm Knee scoring system, *MICP* minimally invasive with cable pin technique, *MIOT* minimally invasive osteosynthesis technique, *MIS* minimally invasive surgery, *NM* not mentioned, *OMATB* open modified anterior tension band technique, *ORTF* open reduction and tension band wiring fixation, *OS* open surgery, *OSKW* conventional open surgery using the K wire tension band method, *POMC* percutaneous osteosynthesis with modified Carpenter’s technique, *PPOS* percutaneous patellar osteosynthesis system, *RCT* randomized controlled trial, *ROM* range of motion; *S*, studied group

### Risk-of-bias assessment

All the four RCTs included had concerns in at least one domain. The major concerns were performance and detection biases, whereby patients, surgeons, and outcome assessors could identify the group to which the patient belonged based on the differences in the operation incision and fixation device between MIPF and ORIF. Additionally, in certain trials [24, 26, 27], patients had to undergo reoperation during the follow-up period. This created a time-varying confounding factor, which further aggravated the concerns related to bias. Random sequence generation was performed in all the trials. Allocation concealment was omitted in one study [27]; thus, its selection bias was graded with some concerns. Regarding attrition and reporting biases, no significant evidence in favor of the study or control group was demonstrated. They were all marked as low risk. Two nonrandomized studies had moderate overall risk of bias. Again, biases due to outcome measurement and deviation from intended intervention was present in these nonrandomized studies, as in the RCTs. One study [25] excluded the lost-to-follow-up patients before analysis. It only presented the final follow-up result despite the study design of a biweekly follow-up in the first month postoperation, then monthly follow-up until the 6th month, and follow-up every 6 months thereafter. Risk of bias existed in patient selection and reported results. The results of methodological quality assessment are presented in Tables 2 and 3.

**Table 2:** Methodological quality assessment of the selected randomized controlled trials

Study, year of publication	Selection bias	Performance bias	Attrition bias	Detection bias	Reporting bias	Overall bias
Shao et al., 2019 [28]	Low risk	Some concerns	Low risk	Some concerns	Low risk	Some concerns
Lin et al., 2015 [27]	Some concerns	Some concerns	Low risk	Some concerns	Low risk	Some concerns
Mao et al., 2013 [26]	Some concerns	Some concerns	Low risk	Some concerns	Low risk	Some concerns
Luna-Pizarro et al., 2006 [24]	Some concerns	Some concerns	Low risk	Some concerns	Low risk	Some concerns

**Table 3:** Methodological quality assessment of the selected nonrandomized studies

Study, year of publication	Bias due to confounding	Bias in selection of participants	Bias in classification of interventions	Bias due to deviations from intended interventions	Bias due to missing data	Bias in measurement of outcomes	Bias in selection of reported results	Overall risk of bias judgment
Vicenti et al., 2020 [10]	Low	Low	Low	Low	Low	Moderate	Low	Moderate
Chiang et al., 2011 [20, 25]	Low	Moderate	Low	Moderate	Low	Moderate	Moderate	Moderate

### Primary outcomes

#### Pain score

**Fig. 2:**
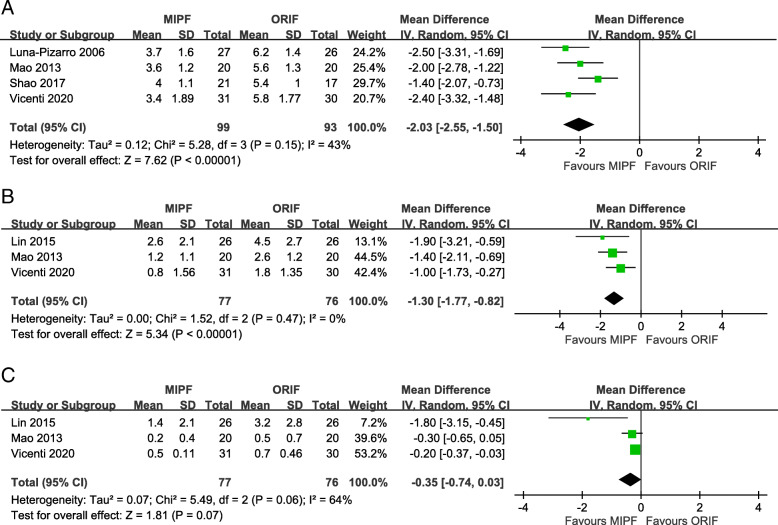
Forest plot of pain scores

In the meta-analysis of VAS, patients undergoing MIPF had statistically more favorable outcomes than those receiving ORIF in the first month postoperation (MD = − 2.03, 95% CI = − 2.55 to − 1.50; *p* < 0.00001; *I*^2^ = 43%). The advantage persisted during the 3-month follow-up (MD = − 1.30, 95% CI = − 1.77 to − 0.82; *p* < 0.00001; *I*^2^ = 0%). No significant difference was observed between the two groups after 6 months (MD = − 0.35, 95% CI = − 0.74 to 0.03; *p* = 0.07; *I*^2^ = 64%). Detailed results are presented in Fig. 2.

#### Range of motion

**Fig. 3:**
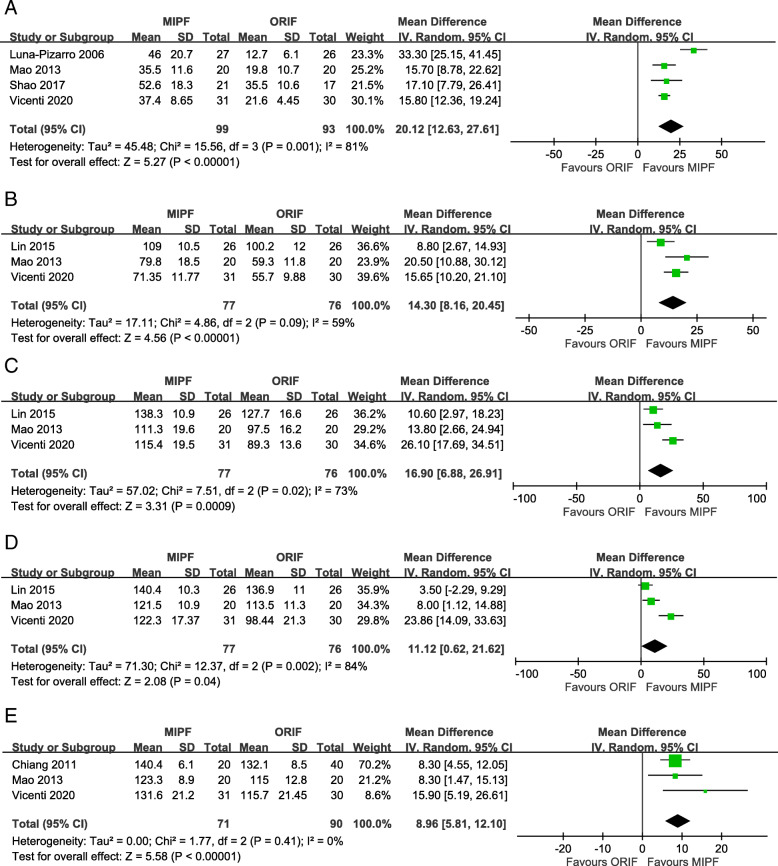
Forest plot of knee flexion angles

Knee flexion angles were significantly higher for MIPF than for ORIF regardless of the follow-up at 1 month, 3 months, 6 months, 12 months, and 24 months (Fig. 3). High heterogeneities in results, however, were observed during the 1st and 12th months. The heterogeneity of results in both groups decreased when SMDs were pooled for estimation. Sensitivity analysis was performed after excluding data from the study by Vicenti et al. [10], the only nonrandomized study with this parameter. Nevertheless, an even higher level of heterogeneity appeared at 1 month (*I*^2^ = 83%), whereas no heterogeneity was observed at 12 months (*I*^2^ = 0%) (Additional file 1). Therefore, the difference in knee flexion remained inconclusive at 1 month in this meta-analysis.

**Fig. 4:**
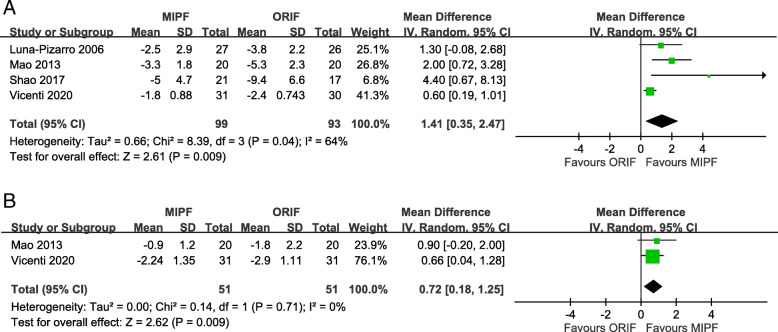
Forest plot of knee extension angles

Similar to knee flexion, knee extension angles were significantly higher for MIPF at 1-month (MD = 1.41, 95% CI = 0.35 to 2.47; *p* = 0.009; *I*^2^ = 64%; Fig. 4a) and 3-month (MD = 0.72, 95% CI = 0.18 to 1.25; *p* = 0.009; *I*^2^ = 0%; Fig. 4b) follow-up. No further analysis is available at 6, 12, and 24 months due to insufficient data.

#### Joint functionality

**Fig. 5:**
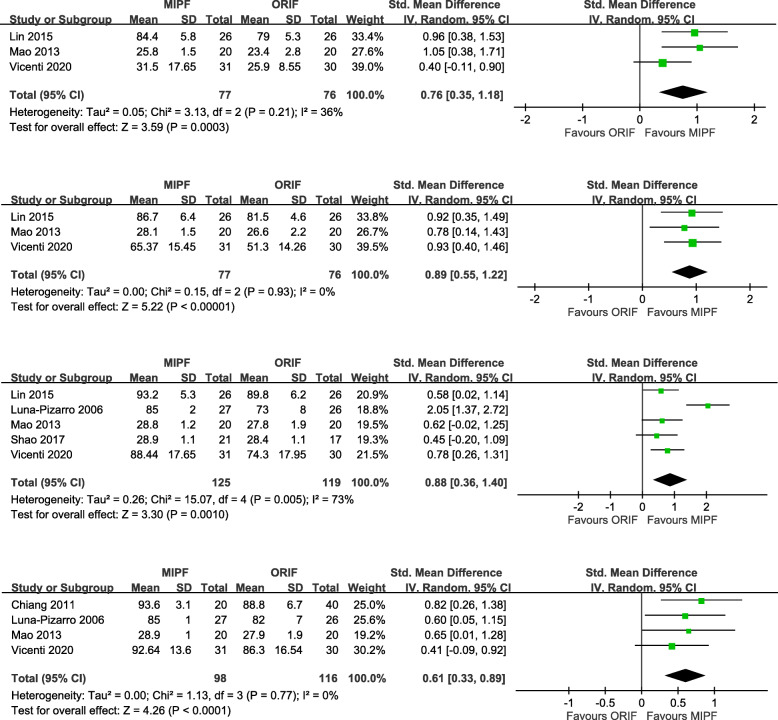
Forest plot of joint functionality

In this meta-analysis, joint functionality was assessed on the basis of the Lysholm knee scoring system, Bostman clinical grading scale, or Knee Society Clinical Rating Scale. The results indicated that, at follow-up, patients who underwent MIPF showed superior joint functionality than those who underwent conventional open surgery. The statistical differences were consistent at 24 months (SMD = 0.54, 95% CI = 0.21 to 0.86; *p* = 0.001). No substantial heterogeneity was found at different time intervals, although a marginal value (*I*^2^ = 73%) (Fig. 5) was observed at 12 months.

### Secondary outcomes

#### Surgical time

**Fig. 6:**
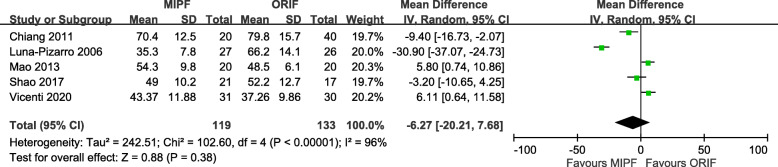
Forest plot of surgical time

Of the six articles included, five measured the surgical time, and the measurements were pooled for calculation. The operative time between MIPF and ORIF was not significantly different, with high heterogeneity (MD = − 6.27, 95% CI = − 20.21 to 7.68; *p* = 0.38; *I*^2^ = 96%) (Fig. 6). Variability was preserved after adjustment of the nonrandomized studies (data not shown).

Subgroup analysis was performed to investigate the underlying causes. However, the operation time was found to be independent of the implantation used during surgery (*p* = 0.29). The *I*^2^ values of both subgroups were still high; an overall aspect other than this component existed. The results of this subgroup analysis are presented in detail (see Additional file 2).

### Complications and implant removal

**Fig. 7:**
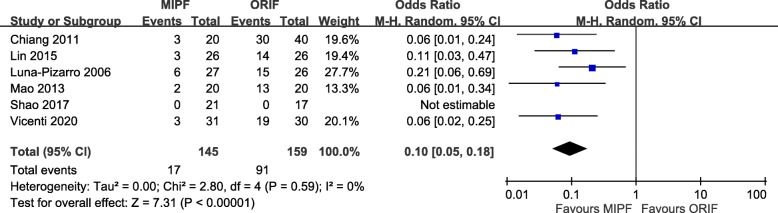
Forest plot of complication rates

Adverse events were recorded in all studies. The risk of complications with MIPF was statistically lower than that with open surgery (OR = 0.10, 95% CI = 0.05 to 0.18; *p* < 0.00001; *I*^2^ = 0%; Fig. 7).

Further meta-analysis of complications showed that displaced fragment and malunion/nonreduction, painful hardware or irritation, loosening or migrating hardware, delayed wound healing, and wire breakage mainly contributed to the differences. No significance was observed in terms of infection. Additional file 3 presents the results of all related complications.

**Fig. 8:**
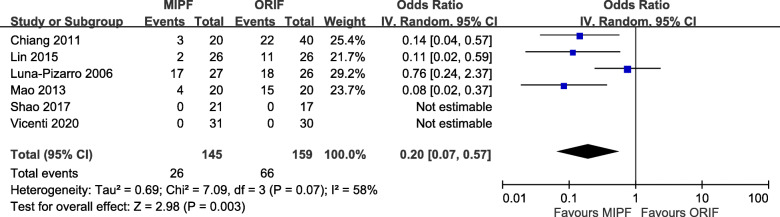
Forest plot of implant removal rates

In response to adverse events, in particular, painful hardware or irritation, patients might have their implant removed. Its incidence was reported in four articles. OR < 1 was obtained in a comparison of the two surgical groups (OR = 0.20, 95% CI = 0.07 to 0.57; *p* = 0.003; *I*^2^ = 58%; Fig. 8).

## Discussion

To the best of our knowledge, this is the first meta-analysis that compared MIPF with ORIF for patella fractures at different time points. Zhang et al. [29] investigated the efficacy of K-wire tension band fixation in comparison with other alternatives, such as cannulated screws, cable pins, and ring pins. That study focused on the differences between distinct fixation materials. Hence, it included studies that did not necessarily compare minimally invasive and open reduction surgeries. Zha et al. [30] compared the cable pin system with K-wire tension band fixation. The results of that study cannot be generalized because the study target was limited to the Chinese Han population, and only parameters at the 6-month follow-up were pooled for analysis.

The minimally invasive procedure is now a modern trend in the medical field. It not only reduces operative trauma but also has more favorable clinical outcomes [31]. Our results revealed that MIPF caused lower pain and had higher extension angles with patella fracture at early follow-up (up to 3 months). Moreover, it was associated with favorable knee flexion angles and functional scores in the long term (for at least 2 years). The advantages are comparable with these in different series and clinical trials [32–35].

The difference between the two procedures in terms of knee extension was minimal relative to knee flexion, which was significant at 2 years. We believe that this result is reasonable. Patella fracture tends to lead to knee stiffness and muscle weakness due to prolonged immobilization for the prevention of secondary displacement or malunion at the beginning. The quadriceps muscle requires time to regain its function. The painful sensation experienced by the patient and the use of an immobilizer, such as brace or splint, further delay the rehabilitation process. MIPF may be beneficial, but after 3–4 months, when the surgical wound heals, physiotherapy determines the difference in outcomes.

SMD was used for the pooled analysis of knee functionality. This statistical technique is used to convert different evaluation scoring systems into one uniform scale. Reasonable comparison can be made even when the given outcome is measured using distinct evaluation tools.

The clinical importance of MIPF is well reflected in our results on knee functionality. With further investigation of the functional scores/scales in each included article, minimal difference may be found at different follow-up periods. However, clinical significance can never be justified based on individual data. Additionally, all the evaluation scoring systems were standardized. Statistical difference and its sustainability provide accurate insights. In this meta-analysis, knee functionality after MIPF was statistically better than that after ORIF up to a period of 2 years. Clinically important variation between the two surgical approaches did exist.

Surgical times between MIPF and ORIF were not significantly different in our analysis. Multiple factors may contribute to this finding. First, MIPF for patella fracture is technically demanding, as surgeons cannot visualize the fracture site straight from where they incise [33, 36]. They have to be familiarized with the use of arthroscopy, fluoroscopy, novel fixation techniques, or other percutaneous osteosynthesis devices. The reduction process can be prolonged when we correct the displacement in a three-dimensional space by using a two-dimensional monitor, examine the fracture site through the lateral view instead of the skyline view, and assess the posterior aspect of the patella in such a small operative scope. Moreover, the physician’s learning curve, frequency of intraoperative imaging, or the surgical difficulty during anatomical reconstruction considerably influence the surgical time. By contrast, MIPF involves a smaller incision. The time for wound closure would be much shorter than that of ORIF [25]. None of our included studies mentioned how they timed the operation. The pros and cons of the two surgical approaches added up to a nonsignificant result.

In this study, the incidence of complications and implant removal with MIPF was low. This merit could be attributed to the improvement of the displaced fragment and malunion/nonunion, painful hardware or irritation, loosening or migrating hardware, delayed wound healing, and wire breakage.

Nonunion is infrequent in patella fractures overall. A meta-analysis reported a patella fracture incidence rate of 1.3% with ORIF [37]. No similar study was performed to quantify the patella fracture incidence rate with MIPF. Thus, MIPF provides a comparable or even lower nonunion rate, although it may not be clinically significant.

For malunion, precise anatomical reduction is crucial. Arthroscopy-assisted MIPF allows surgeons to check for articular congruity through a magnified visual field from inside the joint. Closed reduction through manipulation and fine tuning with clamps could be repeatedly verified through intraoperative imaging, even without arthroscopy. The appropriate height and alignment of the patella undergoing MIPF are warranted. Malunion is uncommon with MIPF compared with the open method [31]. However, these studies had a relatively short follow-up period, which may be inadequate to accurately determine the risk of malunion and its subsequent ramifications, such as posttraumatic osteoarthritis.

Regarding painful hardware or irritation, most studies have not explained the discrepancy between MIPF and ORIF. Gosal et al. [38] suggested that painful hardware is related to the K-wire prominence. Other studies have found that irritation is secondary to implant loosening or migration due to the smooth surface of the K-wire and the method of twisting and bending [12, 39, 40]. The complications are inter-related. Surgical experience has revealed that the soft tissue over our kneecap is subjected to excessive traction during and after ORIF, which prevents wound dehiscence. The stretched skin is predisposed to painful hardware, irritation, and delayed wound healing, which is less with MIPF. From a biomechanical perspective, interfragmentary screw fixation in combination with the tension band principle provides extra stability over the modified tension band wiring or screws alone, regardless of its static or dynamic dimension [41]. This type of fixation is accompanied by additional compression and resistance against the distraction force [42, 43], which in turn gives us some insight on how fixation devices may reduce the risk of complications after MIPF.

Other complications related to MIPF, for example, wound breakdown, neurovascular injury, and osteomyelitis and its concomitant septic arthritis, were not discovered in any of our included articles. Nevertheless, surgeons must be careful in preventing any of them. Of note, the minimally invasive approach is not equivalent to minimal complications, although the occurrence of wound-related complications or any other major complications for patella fractures with MIPF is rare in the literature.

Implant removal is mostly secondary to postoperative complications. Our results showed that the risk of implant removal after MIPF was significantly lower than that after ORIF. This is compatible with what we expected from a reduced complication rate with MIPF in this study.

Various minimally invasive fixation techniques for patella fracture have been proposed to date. Ma et al. [44] reported the use of percutaneous suture with specially made curved and straight needles as well as stainless steel wires. They extended the usage of this method from middle transverse fracture to fractures of the upper or lower poles or comminuted type. Appel et al. [45] presented a surgical procedure of percutaneous pinning without the need for figure-eight wiring. Tandogan et al. [46] applied arthroscopic-assisted reduction and percutaneous cannulated screw fixation. Their techniques were unsuitable for patella fractures with displacement of > 8 mm, as arthroscopic methods cannot completely repair the extensor mechanism. Yannis et al. [47] described the administration of circular external fixation with the help of arthroscopy for comminuted fractures of the patella. The results were satisfactory, although only four patients were studied. Mao et al. [48] performed MIPF with the cable pin system. They later conducted a RCT, which is included in this meta-analysis [26]. All these articles sufficiently demonstrate the benefits of MIPF in treating patella fractures. During study selection, we did not place any restriction on MIPF techniques. Superiority of one technique over the others might be determined when many studies are available for analysis in future.

This meta-analysis has a few limitations. First, due to limited studies on minimally invasive percutaneous osteosynthesis, we found only six relevant studies for the qualitative synthesis. A large number of clinical trials are required for obtaining a comprehensive result. Second, our analysis was limited to the studies published before December 2020. Certain relevant unpublished trials or studies included in databases other than those employed in this meta-analysis may be missed. Third, not all of our included studies were RCTs. The confounding factors might be a cause of bias, although the demographics of the articles were not statistically different between the groups. Fourth, this meta-analysis included different fracture types and fixation devices. No restriction was placed on the implants used or outcome subdivisions for different types of transverse patella fractures. Relevant data should be interpreted with caution, and MIPF efficacy for different fracture patterns with particular instruments may be one direction for further research. Lastly, minimally invasive surgery is not recommended for open or comminuted patella fracture as per today’s clinical standard. Half of the included studies excluded patients with multiple traumas, previous chronic degenerative disease along with its associated fracture, previous surgical intervention of knee, peripheral neural damage, uncompensated diabetes, or severe osteoporosis. Our results may not be applicable to these patients.

## Conclusion

MIPF is a surgical approach that offers lower pain, higher range of motion, higher joint functionality, and lower incidence of complications and implant removal postoperation than ORIF for transverse patella fracture. Therefore, MIPF is a viable alternative to ORIF.

## Supplementary Information

**Additional file 1: Table S1.** Sensitivity analysis of knee flexion angles.

**Additional file 2: Table S2.** Subgroup analysis of surgical time.

**Additional file 3: Table S3.** Results of odds ratios for different complications.

**Additional file 4:.** Funnel plot of pain scores.

**Additional file 5:.** Funnel plot of knee flexion angles.

**Additional file 6:.** Funnel plot of knee extension angles.

**Additional file 7:.** Funnel plot of joint functionality.

**Additional file 8:.** Funnel plot of surgical time.

**Additional file 9:.** Funnel plot of complication rates.

**Additional file 10:.** Funnel plot of implant removal rates.

**Additional file 11:.** Preferred Reporting Items for Systematic Reviews and Meta-Analyses 2009 checklist.

## Data Availability

The datasets used and analyzed during the current study can be obtained from the corresponding author on reasonable request.

## Abbreviations

CI: Confidence interval
MD: Mean difference
MIPF: Minimally invasive percutaneous fixation
OR: Odds ratio
ORIF: Open reduction internal fixation
RCT: Randomized control trial
SMD: Standardized mean difference
VAS: Visual analog scale
