# Supplementary material for: Comparison of minimally invasive percutaneous fixation and open reduction internal fixation for patella fractures: a meta-analysis
Source: J Orthop Surg Res. 2021 Aug 17;16:506. doi: 10.1186/s13018-021-02612-1 (PMC8369684; doi:10.1186/s13018-021-02612-1)
Supplement: Supplementary file 1 — Additional file 1: Table S1. Sensitivity analysis of knee flexion angles. [file 13018_2021_2612_MOESM1_ESM.docx]

Supplementary Information

| **Table, additional file 1.** Sensitivity analysis of knee flexion angles | | | | | | | | | | | | |
| --- | --- | --- | --- | --- | --- | --- | --- | --- | --- | --- | --- | --- |
|  | Before adjustment | | | | | | After adjustment | | | | | |
| Follow-up period | Studies | Participants | MD  [95% CI] | *P* | *I^2^* | References | Studies | Participants | MD  [95% CI] | *P* | *I^2^* | References |
| 1 month | 4 | 192 | 20.12  [12.63 to 27.61]^a^ | <0.00001 | 81% | 10. 24, 26, and 28 | 3 | 131 | 22.01  [10.77 to 33.25]^a^ | 0.0001 | 83% | 24, 26, and 28 |
| 12 months | 3 | 153 | 11.12  [0.62 to 21.62]^a^ | 0.04 | 84% | 10, 26, and 27 | 2 | 92 | 5.37  [0.94 to 9.80]^a^ | 0.02 | 0% | 26 and 27 |
| CI, confidence interval; MD, mean difference  ^a^A significant difference exists in the comparison of the two groups | | | | | | | | | | | | |
