# Supplementary material for: Comparison of minimally invasive percutaneous fixation and open reduction internal fixation for patella fractures: a meta-analysis
Source: J Orthop Surg Res. 2021 Aug 17;16:506. doi: 10.1186/s13018-021-02612-1 (PMC8369684; doi:10.1186/s13018-021-02612-1)
Supplement: Supplementary file 2 — Additional file 2: Table S2. Subgroup analysis of surgical time. [file 13018_2021_2612_MOESM2_ESM.docx]

| **Table, additional file 2.** Subgroup analysis of surgical time | | | | | | |
| --- | --- | --- | --- | --- | --- | --- |
| Fixation device | Studies | Participants | MD  [95% CI] | *P* | *I^2^* | References |
| Zimmer cable pin system | 2 | 78 | 1.73  [-7.05 to 10.51] | 0.70 | 74% | 26 and 28 |
| Others  (PPOS, stainless steel wire or cannulated screws with wires) | 3 | 174 | -11.37  [-33.92 to 11.17] | 0.32 | 97% | 10, 24, and 25 |
| Subgroup differences |  |  |  | 0.29 | 11.3% |  |
| CI, confidence interval; MD, mean difference; PPOS, percutaneous patellar osteosynthesis system | | | | | | |
