# Supplementary material for: Comparison of minimally invasive percutaneous fixation and open reduction internal fixation for patella fractures: a meta-analysis
Source: J Orthop Surg Res. 2021 Aug 17;16:506. doi: 10.1186/s13018-021-02612-1 (PMC8369684; doi:10.1186/s13018-021-02612-1)
Supplement: Supplementary file 3 — Additional file 3: Table S3. Results of odds ratios for different complications. [file 13018_2021_2612_MOESM3_ESM.docx]

| **Table, additional file 3.** Results of odds ratios for different complications | | | | |
| --- | --- | --- | --- | --- |
| Complications | OR  [95% CI] | *P* | *I^2^* | References |
| Displaced fragment and mal/non-reduction | 0.25  [0.07 to 0.95] ^a^ | 0.04 | 0% | 24, 25, 26, and 27 |
| Infection | 0.23  [0.04 to 1.41] | 0.11 | 0% | 24, 25, and 27 |
| Painful hardware and irritation | 0.25  [0.12 to 0.51]^a^ | 0.0002 | 0% | 24, 25, 26, and 27 |
| Loosening and migration | 0.06  [0.01 to 0.34]^a^ | 0.001 | 0% | 25, 26, and 27 |
| Delayed wound healing | 0.1  [0.01 to 0.84]^a^ | 0.03 | 0% | 10 and 26 |
| Broken wires | 0.28  [0.09 to 0.95]^a^ | 0.04 | 0% | 10, 25, and 26 |
| Others (scars, keloid, nonunion, refracture, and tendon tear) | 0.11  [0.01 to 0.86]^a^ | 0.04 | 0% | 10 and 25 |
| CI, confidence interval; OR, odds ratio  ^a^A significant difference exists in the comparison of the two groups | | | | |
