# Supplementary figures and images for: Comparison of minimally invasive percutaneous fixation and open reduction internal fixation for patella fractures: a meta-analysis
Source: J Orthop Surg Res. 2021 Aug 17;16:506. doi: 10.1186/s13018-021-02612-1 (PMC8369684; doi:10.1186/s13018-021-02612-1)

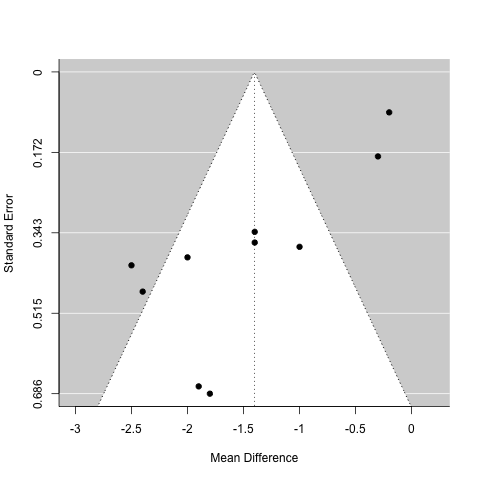

Supplement: Supplementary file 4 — Additional file 4:. Funnel plot of pain scores. [file 13018_2021_2612_MOESM4_ESM.docx]

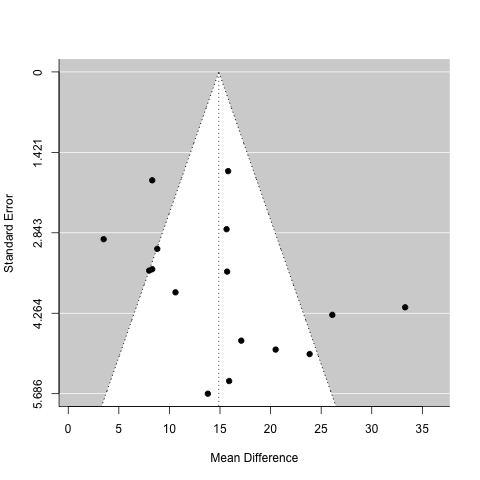

Supplement: Supplementary file 5 — Additional file 5:. Funnel plot of knee flexion angles. [file 13018_2021_2612_MOESM5_ESM.docx]

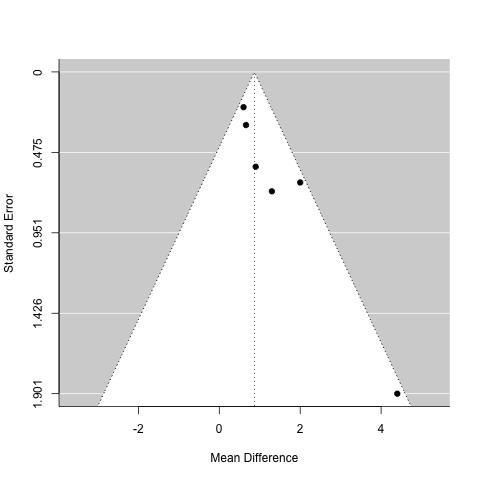

Supplement: Supplementary file 6 — Additional file 6:. Funnel plot of knee extension angles. [file 13018_2021_2612_MOESM6_ESM.docx]

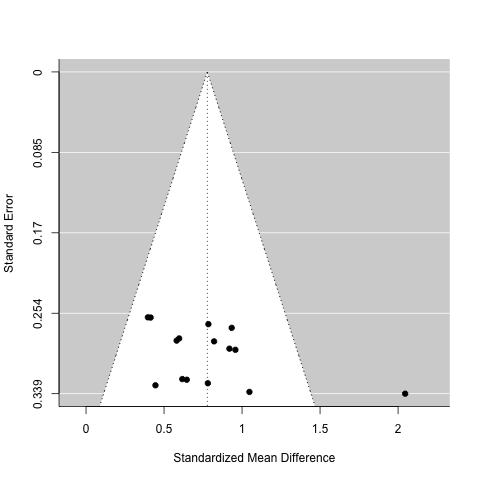

Supplement: Supplementary file 7 — Additional file 7:. Funnel plot of joint functionality. [file 13018_2021_2612_MOESM7_ESM.docx]

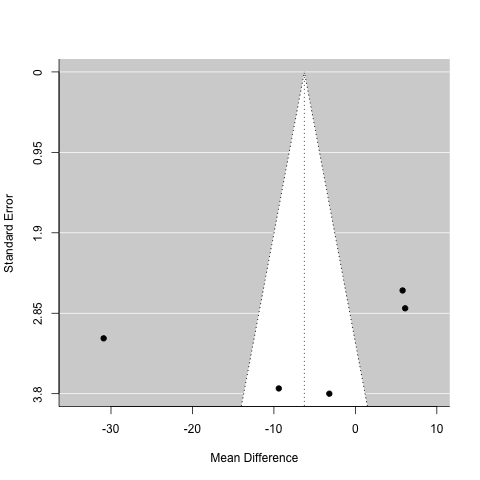

Supplement: Supplementary file 8 — Additional file 8:. Funnel plot of surgical time. [file 13018_2021_2612_MOESM8_ESM.docx]

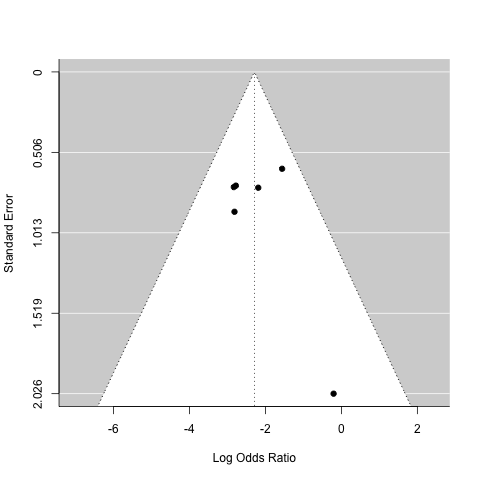

Supplement: Supplementary file 9 — Additional file 9:. Funnel plot of complication rates. [file 13018_2021_2612_MOESM9_ESM.docx]

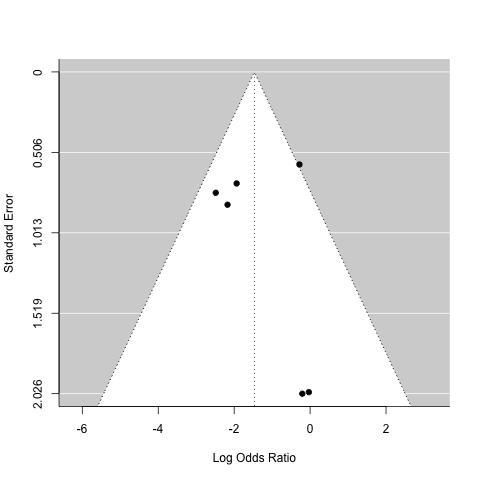

Supplement: Supplementary file 10 — Additional file 10:. Funnel plot of implant removal rates. [file 13018_2021_2612_MOESM10_ESM.docx]
